# The Clinical Benefits and Accuracy of Continuous Glucose Monitoring Systems in Critically Ill Patients—A Systematic Scoping Review

**DOI:** 10.3390/s17010146

**Published:** 2017-01-14

**Authors:** Sigrid C. J. van Steen, Saskia Rijkenberg, Jacqueline Limpens, Peter H. J. van der Voort, Jeroen Hermanides, J. Hans DeVries

**Affiliations:** 1 Clinical Diabetology, Academic Medical Center, P.O. Box 22660, 1100 DD Amsterdam, The Netherlands; j.h.devries@amc.uva.nl; 2 Department of Intensive Care Medicine, Onze Lieve Vrouwe Gasthuis, P.O. Box 95500, 1090 HM Amsterdam, The Netherlands; s.rijkenberg@olvg.nl (S.R.); p.h.j.vandervoort@olvg.nl (P.H.J.v.d.V.); 3 Medical Library, Academic Medical Center, P.O. Box 22660, 1100 DD Amsterdam, The Netherlands; c.e.limpens@amc.uva.nl; 4 Department of Anesthesiology, Academic Medical Center, P.O. Box 22660, 1100 DD Amsterdam, The Netherlands; j.hermanides@amc.uva.nl

**Keywords:** (blood) glucose, continuous glucose monitoring (CGM), glucose sensors, biosensing techniques, intensive care (unit), critical illness, accuracy

## Abstract

Continuous Glucose Monitoring (CGM) systems could improve glycemic control in critically ill patients. We aimed to identify the evidence on the clinical benefits and accuracy of CGM systems in these patients. For this, we performed a systematic search in Ovid MEDLINE, from inception to 26 July 2016. Outcomes were efficacy, accuracy, safety, workload and costs. Our search retrieved 356 articles, of which 37 were included. Randomized controlled trials on efficacy were scarce (*n* = 5) and show methodological limitations. CGM with automated insulin infusion improved time in target and mean glucose in one trial and two trials showed a decrease in hypoglycemic episodes and time in hypoglycemia. Thirty-two articles assessed accuracy, which was overall moderate to good, the latter mainly with intravascular devices. Accuracy in critically ill children seemed lower than in adults. Adverse events were rare. One study investigated the effect on workload and cost, and showed a significant reduction in both. In conclusion, studies on the efficacy and accuracy were heterogeneous and difficult to compare. There was no consistent clinical benefit in the small number of studies available. Overall accuracy was moderate to good with some intravascular devices. CGM systems seemed however safe, and might positively affect workload and costs.

## 1. Introduction

Stress-induced hyperglycemia occurs in over 90% of the patients admitted to an Intensive Care Unit (ICU), irrespective of a previous diagnosis of diabetes [1]. This hypermetabolic state is a response to severe illness and results from increased gluconeogenesis, enhanced peripheral insulin resistance and beta-cell secretory defects, due to a complex interaction between excessive counter regulatory hormones and cytokines [2]. This phenomenon was regarded as physiological, although several studies showed an association between hyperglycemia and mortality in critically ill patients [3,4,5,6]. Likewise, hypoglycemia and glycemic variability were shown to relate to adverse outcomes [7]. Although the mechanism by which dysglycemia results in adverse clinical outcomes is not fully understood, these findings have highlighted the importance of glucose control. In 2001, van den Berghe and colleagues were able to show a substantial mortality benefit in a surgical ICU when intensive insulin therapy (IIT) was used to treat hyperglycemia to target a glucose between 80 and 110 mg/dL [1]. Several trials on IIT have been conducted since then, but the initial beneficial effects could not be confirmed [8,9,10,11,12,13]. In addition, there was an increased risk for hypoglycemia associated with IIT in these trials, with 5.1% to 18.7% of patients experiencing one or more episodes of severe hypoglycemia [1,8,12], which is accompanied by a ±2 fold increased mortality risk [14,15]. Due to differences in study populations, pursued target ranges and measurement devices, it is difficult to compare results and draw definite conclusions. Nevertheless, recent meta-analyses did not show a beneficial effect of tight glycemic control on mortality [16,17,18]. Nowadays, consensus states that hyperglycemia in critically ill patients should be monitored and treated, with guidelines recommending glucose levels between 100 and 150 mg/dL (Society of Critical Care Medicine [19]) or between 140 and 180 mg/dL (American Diabetes Association [20]). However, tighter ranges might be feasible when the average ICU has the ability to safely control glucoses in such a range [21]. 

Currently, glucose control in the ICU is mostly based on intermittent measurements with handheld meters for point-of-care glucose testing. These periodic measurements are used to guide intravenous insulin administration based on a (local) algorithm. Handheld glucose meters are not designed for ICU use, and their accuracy is questionable and markedly inferior to central laboratory or blood gas analysis, especially in patients with anemia, hypoxia or when exposed to certain drugs [22,23]. Moreover, the intermittent character makes it impossible to observe important glucose fluctuations. In critically ill patients, between 4% and 15% of hypoglycemic events are undetected [24] and hypoglycemic episodes occur more frequently when there is a longer time interval between glucose measurements [25]. Continuous Glucose Monitoring (CGM) systems provide (near-) continuous information about glucose levels, thereby creating the possibility to detect acute changes and real-time trend data, and improve the quality and efficiency of glucose control. Moreover, they could decrease the time spent on achieving glycemic control, since tight glycemic control is burdensome and reported to take almost 1.5 to 2 h of a 24 h single patient nursing period [26,27]. Considering the impact of frequent blood glucose monitoring in the critically ill, continuous systems could be advantageous by improving glycemic control and reduce the burden on (nursing) staff. 

There are different CGM devices available, employing various measurement techniques (glucose oxidase, mid-infrared spectroscopy or fluorescence) with positioning in either the interstitial (minimal invasive) or intravascular (invasive) space [28]. One noninvasive transdermal device (Symphony, Echo Therapeutics), is claimed to be under investigation. Devices can be labeled as ‘continuous’ when they have a measurement or sampling frequency of at least once every 15 min or more frequently [29]. The use of subcutaneous systems is already accepted in the outpatient setting, where a high accuracy is shown as compared to reference blood glucose measurement [30]. CGM systems have been evaluated over almost 10 years in the ICU. Although the use of a CGM system, especially when combined with an appropriate insulin dosing protocol, has the potential to improve efficient and safe glucose control, it is still not common practice in the ICU. Moreover, accepted standardization of metrics to evaluate the benefit of CGM systems was lacking, until an ICU expert consensus statement came out [31]. With this systematic scoping review, we aimed to assess the evidence regarding the clinical benefits and accuracy of CGM systems in critically ill patients.

## 2. Materials and Methods

### 2.1. Eligibility Criteria

For this review, we included articles that reported original empirical data on the use of a CGM system (located subcutaneous or intravascular) in critically ill patients, admitted to an ICU. Outcome measures of interest were efficacy, accuracy, adverse events, workload and costs. To estimate efficacy we included only randomized controlled trials (RCTs). Assessment of efficacy in these trials had to at least cover one metric of the average glucose level or time spent in different glucose ranges. The CGM system had to be compared to standard of care or head-to-head with another CGM system. Articles were excluded when they did not, or not explicitly, report the outcomes of interest, when the CGM system had an sampling interval over 15 min (since this is considered as non-continuous [29]), or when the patients studied were considered to be not representative for the general ICU population (e.g., highly specific patient groups, such as (premature) neonates and patients undergoing pancreatic surgery). Studies with the STG closed-loop system by Nikkiso were excluded since this system is not a CGM system, but an artificial pancreas system that is only available in Japan [32]. To assess the accuracy we included both RCTs and observational studies. Accuracy studies were considered eligible when the investigational CGM system output was compared to an arterial or venous reference sample (since capillary reference measurements are considered inaccurate) [22]. To be eligible, at least two of the following accuracy outcome measures had to be reported (for overview see Appendix B): Mean (or median) Absolute Relative Difference (MARD), Clarke Error Grid (CEG [33]), (modified) Bland-Altman plot [34] or agreement with the accuracy standards of the International Organization for Standardization (ISO) [35,36].

### 2.2. Search Methods

A medical information specialist (J.L.) performed an electronic search in Ovid MEDLINE (including Epub Ahead of Print, In-Process & Other Non-Indexed Citations, Ovid MEDLINE(R) Daily and Ovid MEDLINE(R)) from inception to 26 July 2016. The search strategy consisted of MesH terms and text words for the concepts CGM (including continuous or real time glucose, glucose sensor, glucose monitor and specific CGM devices) and critically ill patients (including (pediatric) intensive care, burn center, coronary care). No methodological search filter and language or date restrictions were applied. Animal studies were safely excluded by double negation (not (animals/not humans)) (see Appendix A for entire MEDLINE search strategy). Additionally, we applied forward and backward snowballing of identified relevant articles.

### 2.3. Study Selection

Two independent authors (C.J.S. and S.R.) screened title and/or abstract of the articles against the in- and exclusion criteria and included appropriate articles based on their full text. In case of doubt or disagreement about the inclusion, a third author (J.H.D.) was involved, and consensus was reached by discussion.

### 2.4. Data Extraction and Handling

Data extraction was independently performed by two authors (S.C.J.v.S. and S.R.) based on a predefined form. For each eligible article, the following data items were extracted: publication year, study design, type and number of included patients, in- and exclusion criteria, intervention and control, reference method, baseline characteristics, local glucose protocol and target ranges, outcome measures and authors conclusions. Discrepancies were resolved through consensus. We described the main results of the study qualitatively and in evidence tables. 

## 3. Results

### 3.1. Results of the Search

**Figure 1:**
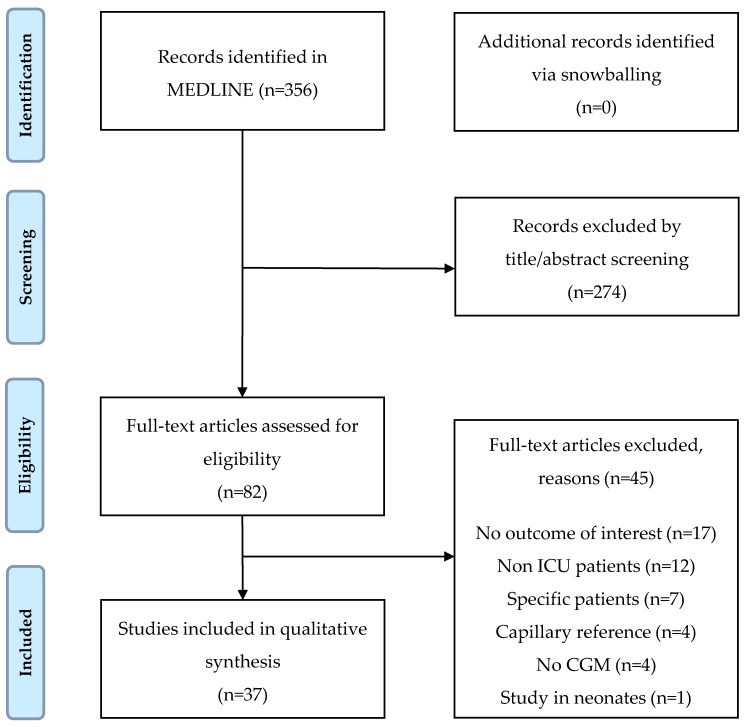
Flow diagram of study selection.

The MEDLINE search retrieved 356 unique citations. Snowballing did not yield additional publications. We excluded 274 citations on the basis of title and/or abstract and screened 82 articles full text. Of these, 37 articles met our in- and exclusion criteria and were included in this review. The study selection process and reasons for exclusion are presented as a flow diagram (Figure 1). 

### 3.2. Study Descriptives

Table 1 provides an overview of the 37 articles included in this review, sorted by their main outcome (efficacy versus accuracy). Five RCTs primarily assessed the efficacy of subcutaneous CGM systems [37,38,39,40,41]. We found no RCTs on intravascular devices. Accuracy was the main outcome of the other 32 articles, with a minority focusing on intravascular devices. Almost half of the articles studied a mixed ICU population, but 9 (28%) of 32 accuracy studies were restricted to cardiac surgery patients. Four observational studies concerned children [42,43,44,45]. CGM systems were studied for a median of 72 h, with a maximum of 7 days. Overall, the number of analysed patients varied between 8 and 174, with a median of 24 patients. Most studies used arterial or both arterial and venous samples as reference; four studies used only venous reference samples. 

### 3.3. Efficacy

We identified 5 RCTs, conducted between 2010 and 2015, that investigated the efficacy of subcutaneous CGM systems on glycemic control (Table 2). One trial studied a microdialysis based system (GlucoDay, A. Menarini Diagnostics), the other devices were based on an electrochemical electrode technique (FreeStyle Navigator, Abbott and Guardian REAL-Time, Medtronic). The sample size ranged from 24 to 156 patients. Study duration varied between 24 h and 5 days. One study included only cardiac surgery patients [40], the other trials included ICU patients with various medical or surgical conditions. All trials included both patients with and without preexisting diabetes (percentage diabetes patients 25%–40%). The CGM systems used different approaches to guide treatment. De Block and colleagues used high rates of glucose change (>25 mg/dL per 30 min) to prompt the nursing staff to take extra arterial reference samples in a group of patients with a high APACHE score (mean 28 ± 7) [37]. In the control patients (*n* = 19) CGM readings were blinded. These reference samples were used to adjust intravenous insulin dose based on a modified Yale protocol. The CGM device in the intervention group (*n* = 16) was calibrated 6 times in 48 h as compared to 2 times in 48 h in the control group (the latter following manufacturer’s instructions). The use of this CGM system did not improve mean glucose (intervention 119 ± 17 mg/dL versus control 122 ± 11 mg/dL, not significant (NS) (actual *p*-value not reported), time in target (intervention 37% ± 12% versus control 34% ± 10%, NS), or glycemic variability (NS). Although not significant, the time in hypoglycemia (intervention 9 ± 23 min per 24 h versus control 35 ± 62 min per 24 h, NS) as well as the number of patients with hypoglycemic events (intervention 3 versus control 9, NS) was considerably lower in the intervention group. Likewise, adjustment of the insulin infusion rate by regularly inserting CGM readings into an insulin advising algorithm (every 15 min by Kopecky [40], and every 2 h by Holzinger [41]), did not improve mean glucose or time in range, defined as 80–110 mg/dL by Kopecky and <110 mg/dL by Holzinger. Both studies did show lower (severe) hypoglycemia rates in the CGM intervention group, in the latter with a hypoglycemia rate of 1.6% in the intervention and 11.5% in the control group, giving a 9.9% absolute risk reduction (95% CI 1.2–18.6, *p* = 0.031). Boom et al. used the alarms of the CGM system, set at glucose <90 mg/dL or >162 mg/dL, to enter additional glucose values into an insulin advising algorithm (designed for intermittent measurements) in two groups of 78 patients [38]. As compared to usual care with blinded CGM this did not increase time in target (intervention 69% ± 26% versus control 66% ± 26%, *p* = 0.47). When combining the CGM system with automated closed-loop insulin therapy in mainly neurosurgical patients, as done by Leelarathna et al., mean glucose was significantly reduced and time in target improved with over 35% (intervention 54.3% [44.1–72.8] versus control 18.5% [0.1–39.9], *p* = 0.001), especially within the first 24 h, and this effect was persistent when ranges were widened [39]. There were no hypoglycemic events (<72 mg/dL) and there was no significant between group difference in the amount of insulin administered. However, in the intervention and control group different target ranges were used (intervention 108–144 mg/dL versus control 126–180 mg/dL) and the intervention group had a higher frequency of calibration. Thus, with this closed-loop system a lower and smaller target glucose range could be achieved, without inducing the risk for hypoglycemia. We did not perform a meta-analysis on these trials given the (clinical) heterogeneity introduced by different devices, study populations and glucose targets. 

### 3.4. Accuracy

The accuracy of CGM systems was assessed in all included articles, with 26 articles investigating subcutaneous systems (Table 3) and 11 investigating intravascular systems (Table 4). The relevant accuracy metrics are explained in Appendix B. Four studies used a blood gas analyzer for reference measurements, but did not specify the sample location [43,45,46,47]. In all other studies the reference was specified as arterial and/or venous. 

**MARD.** The Mean Absolute Relative Difference (MARD) quantifies the deviation from the reference measurement. A lower MARD corresponds with better accuracy. MARD varied widely among studies. With regard to the subcutaneous devices, the highest reported MARDs were 30.5% with the FreeStyle Libre (Abbott Diabetes) [48], and 23.2%–23.7% with the Guardian REAL-Time, (Medtronic) depending on location (thigh or abdomen) [49]. Excluding these outliers, MARD ranged from 7% to 15.6% (both with the FreeStyle Navigator [50]). The needle-free, transdermal device, the Symphony, showed a MARD of 12.3% [51]. Intravascular devices showed overall lower MARD values, ranging between 5.1% (GlucoClear, Edwards Lifesciences [52]) and 14.2% (GluCath, GluMetrics [53]). When comparing arterial (63 sensors) with venous (9 sensors) positioning, as done by Strasma et al., arterial resulted in lower MARD values (arterial 9.6% versus venous 14.2%) [53]. It has to be noticed that intravascular devices can be placed either in the central venous or peripheral (venous or arterial) circulation. Peripherally placed devices suffer more from hypothermia, movements and vasospasm, which can impair accuracy of the device. 

**ISO.** The ISO guideline described the accuracy requirements for intermittent self-monitoring devices in order to achieve regulatory approval but is also used to assess CGM accuracy. Most studies used the ISO 2003 criteria, accepting a 20% bias from the reference measurement for glucose levels >75 mg/dL (and 15 mg/dL bias when glucose level <75 mg/dL) [35]. The stricter guideline from 2013 accepts a 15% bias when glucose levels are >100 mg/dL, and 15 mg/dL when glucose is <100 mg/dL [36]. The proportion of subcutaneous CGM readings within 20% of the reference value was between 68.1% and 94.0%, with exception of the FreeStyle Libre [48], which showed only 7.0% of the CGM readings within 20% of the reference. Intravascular devices had overall higher ISO agreement, with even up to 100% with the Eirus system (Maquet Critical Care) [54]. Stricter than the ISO requirement, the ICU expert consensus states that it is desirable to have 98% of readings within 12.5% of the reference standard [31]. This was reported in two studies [55,56], and with 60.3% and 58.0% within the 12.5% zone these devices did not meet this criterion. 

**Clarke Error Grid (CEG).** CEG analysis indicates the clinical accuracy of the CGM system by connecting the imprecision of the device to the therapy implications [33]. A CEG analysis was performed in 29 (78%) out of 37 studies, and all used grid glucose target between 70 mg/dL and 180 mg/dL, as originally described by Clarke. Two subcutaneous devices had 100% of the paired samples in the acceptable zones A and B (DGMS, San Meditech [57] and CGMS System Gold, Medtronic [58]). In these studies, only a few points were in the hypoglycemic range (exact numbers not reported). All other studies had a minority of samples in possibly dangerous zones. All but one of the studies that reported a CEG of intravascular devices showed 100% to be in zone A and B [46,52,54,59,60]. 

**Bland-Altman.** Bland-Altman plots show the mean bias and limits of agreement (1.96 × standard deviation) between CGM reading and reference measurement, indicating the systematic and random errors. Both subcutaneous and intravascular devices showed generally low mean bias, but there were outliers, with bias in intravenous devices ranging from −10.8 mg/dL to 4.1 mg/dL, and in subcutaneous devices from −43.2 mg/dL to 14.9 mg/dL. Limits of agreement were overall high, as can be seen from Table 3 and Table 4. 

**Intravascular versus subcutaneous.** One study made a head-to-head comparison between the Eirus intravascular CGM system and the subcutaneous FreeStyle Libre, showing the latter to be by far inferior in terms of accuracy (MARD of 30.5% versus 6.5%) [48]. Another study, comparing the intravascular GluCath with the subcutaneous FreeStyle Navigator in 8 patients reported similar accuracy between the two devices in terms of MARD, ISO agreement and Bland-Altman analysis [61]. 

**Effect of calibration.** Leelarathna et al. investigated the effect of calibration frequency on accuracy outcomes in a subcutaneous device (FreeStyle Navigator) [50]. The intervention group calibrated at variable intervals of 1 to 6 h, on average 9.5 times in the first 24 h, and 7 times in the second 24 h. They were compared to a calibration frequency of 4 times in the first 24 h and no calibrations in the second 24 h (following manufacturer’s instructions). Enhanced calibration resulted in a significant lower MARD (intervention 7.0% [3.5, 13.0] versus control 12.8% [6.3, 21.8], *p* < 0.001), more points in zone A of the CEG (intervention 87.8% versus control 70.2%) and higher agreement with the ISO criteria (70.2% versus 87.8%). Three other studies investigated the effect of calibration frequency on accuracy. Van Hooijdonk et al. used routinely obtained blood glucose measurement as additional calibrations, resulting in a mean increase of 6 times in contrast to the requested calibrations [56]. They showed that the number of calibrations had a positive effect on accuracy. With each additional calibration, the absolute difference between CGM reading and reference decreased with 1.4%. In the study by Yue and colleagues, there was significant improvement in MARD when comparing calibration within 6 h with calibration between 6 and 12 h (8.8% ± 7.2% versus 20.1% ± 13.5%, *p* < 0.0001) [57]. The same was seen for data points in zone A of the CEG (92.4% versus 57.1%, *p* < 0.0001). De Block reported that the data points in zone A and B increased from 95% to 97%, when calibration frequency of the GlucoDay went from 2 to 6 times per day, with fewer points in zone C (4.5% versus 1.6%) [62]. 

**Factors influencing accuracy.** Five studies tried to identify factors that could influence accuracy. Accuracy was not influenced by use of norepinephrine [63], or dependent on reason of admission (medical versus surgical) [64]. In the study by Kosiborod et al., MARD was equal between patients with a high and low cardiac surgery risk score [65]. It did however deteriorate in diabetes patients [56,66] and with use of vasopressors, higher SOFA scores, glycemic variability and in the hyperglycemic range [55]. Septic status seemed to improve accuracy [64]. There was no evident difference between various sensor locations (abdomen, thigh, shoulder) [49]. Microcirculation, measured using a microvascular flow index, had no effect on accuracy [66]. None of the studies discussed the use of acetaminophens, although its influence on CGM accuracy is well known from studies in the outpatient setting [67].

### 3.5. Safety

In 14 (40%) of 35 studies there was no description on the occurrence of adverse events. In the studies that did report adverse events, no serious adverse events occurred. Complications of the subcutaneous device were minor bleeding after insertion in 4 (20%) of 20 patients [55], bruises in 13 (13%) of 102 sensors and redness in 13 (13%) of 102 sensors [56]. One study reported a thrombus rate (on ultrasound) of 21% in arterial devices and even of 66% in venous devices, with two complete venous occlusions that required treatment, one of which turned out to be device related thrombosis [53]. Macken et al. and Crane et al. reported both cases of thrombus formation on ultrasound with two intra-arterial catheters (GluCath, Glumetrics and GlySure, GlySure), but no treatment was required [60,68].

### 3.6. Workload and Costs

Boom et al. were the only one to investigate the effect of a CGM, the FreeStyle Navigator I, on costs in a 24 h timeframe [38]. Their analysis showed a mean 12 euro benefit in favor of the CGM system (95% CI 5 to 22 euro, *p* = 0.02). This profit came mainly from the reduction in nursing time and the decline in cost due to laboratory and POC measurements. They also calculated the effect on nursing workload and showed a mean reduction of 19 min per 24 h (intervention 17 min, control 36 min, *p* < 0.001). Wollersheim and colleagues used questionnaires to assess the nurse feedback [55]. During the study they were assigned with observing the trend line and performing additional BG measurements, and not with inserting the sensor or performing calibrations. With a 1/3 response rate, almost 80% of the nurses rated the subcutaneous Sentrino CGM system (Medtronic) as not beneficial and more than half of the nurses described disadvantages of the system, mainly inadequate alarms performance (mentioned in 23.3% of the replies). This is in accordance with findings by Kosiborod et al., who showed high false alarm rates with the Sentrino for both hypoglycemia (70.2%) and hyperglycemia (53.5%) [65]. In contrast, the latter reported better nurse acceptance with the same device, with a 100% positive opinion on performance after using it in two patients. 

### 3.7. Children

Accuracy in critically ill children was assessed in four observational studies [42,43,44,45]. Overall, accuracy seemed lower in children than in adults. MARD ranged from 15.3% [43] to 23.0% [44]. Like in the above studies, most data points were in zone A and B of the Clarke error grid, with exception of the trial by Prabhudesai et al., which had 7.2% of data in zone D [45]. This study also showed high mean bias in the Bland-Altman plot. There were no adverse events reported. 

## 4. Discussion

This systematic scoping review evaluated 37 articles that included both RCTs and observational studies. The majority of the studies were single center studies with modest sample sizes. Study duration was in general short, with an average of 72 h. In addition, the number of RCTs was small (*n* = 6). Although the number of studies increased over the years, the heterogeneity in study populations, interventions and reported outcomes impeded us to draw general conclusions. Moreover, the fact that studies were performed in settings with different local standards of care probably had an important effect on outcomes. 

Overall, in terms of efficacy, the use of subcutaneous CGM systems does not seem to improve the glycemic control of critically ill patients convincingly in a clinically significant manner. With regard to this conclusion, it has to be taken into account that RCTs were scarce (*n* = 5) and that the included trials showed methodological shortcomings to a greater or lesser extent. Even for the two largest RCTs it could be argued that they lacked the appropriate sample size to conclude on their secondary glycemic outcomes. In the trials by Boom and Holzinger the difference in mean sensor glucose was on the border of statistical significance favoring the group receiving a CGM system, with *p*-values of respectively 0.07 and 0.076. Although the 95% confidence intervals should have been reported, the differences in mean glucose of 0.3–0.4 mmol/L were small, with unclear clinical significance.

Moreover, the RCTs used the readings of the CGM system in different manners. Two studies used the CGM as a prompt to obtain additional arterial samples [37,38]. This approach will detect important changes between intermittent measurements, but does not fully use the advantages of the continuous measurements. In the study by de Block, the nurses had to notice high rates of change, without help from an alarm function, since the GlucoDay is not equipped with an alarm function. The *p*-values in this study were denoted as non-significant, but the actual *p*-values were not reported. The trial by Kopecky suffers from the same methodological flaw. Two other studies assigned their nurses to regularly use CGM reading as additional input to the local algorithm [40,41]. This might have led to bias as alarm functions were not used and the input was therefore dependent on nurse adherence to the protocol. In addition, this compliance to the protocol was not measured or reported in these trials. Moreover, this approach might even increase workload, instead of reducing it. The only study that evidently showed improved glycemic control used a fully automated closed-loop system and an adapted glucose algorithm [39]. In the included trials, hypoglycemic events occurred in 11.5% of patients [41] to 2.4% of time [37]. In three of the five RCTs there was an overall reduction in the number of hypoglycemic episodes, as well as the time in hypoglycemia in the CGM group [37,40,41], but this did not reach statistical significance. Hypoglycemia rate is highly dependent on local glucose targets, which varied in the different trials. When the treatment target range is increased, which was done after publication of the NICE-SUGAR results, the hypoglycemia rate diminishes substantially [69]. Comparability of results would benefit from greater consistency in reporting and consensus on outcome measures, as for example stated in a consensus report for artificial pancreas studies in outpatients [70], To show an improvement in glycemic control with CGM systems is difficult, since most ICUs already successfully maintain adequate glucose control, with adequate mean glucose and time in range, and low rates of hypoglycemia with the current standard of care. To take full advantage of CGM devices, the glucose control algorithm probably needs to be adapted to the continuous measurements, which was usually not done in the included studies, except for the closed-loop system. Thus, the number of RCTs on this topic, as well as the sample sizes, are relatively small, which makes it difficult to draw firm conclusions. There are no RCTs conducted that investigated intravascular devices, so we are not able to conclude on the clinical benefits of intravascular systems in the ICU. 

Accuracy is based on comparison between sensor and simultaneously obtained reference values. There are multiple assessment methods, but there is no consensus yet for determining and reporting CGM accuracy, as can be seen from the different methods in which accuracy was assessed and reported. The current expert consensus recommendations on reporting state that a MARD <14% is acceptable and that 98% of the readings >100 mg/dL should be within 12.5% and the remaining 2% within 20% of the reference measurement ([31], Appendix B). The consensus statement does not differentiate between pooled MARD of all data points and individual MARDs. By requiring a pooled MARD to be below a certain cut-off, patients with substantially higher MARDs may go unnoticed. In our opinion, it would be advisable to also set a requirement for the dispersion around the average MARD. The third consensus recommendation is that all data pairs should ideally be in zone A of the CEG. None of the included studies meets all these recommendations. Moreover, not all articles describe the information required, such as nature of reference blood sampling and measurement technology. Some studies used reference measurements from multiple sources, which can be seen as a weakness in design. To assess accuracy, time-matched sensor-reference pairs are used by all included studies. These time-matched points are statistically matched, but might be sub optimally matched from a physiological point of view, because of the difference in glucose concentration between compartments depending upon the glucose rate of change (time lag), which is especially a problem with subcutaneous devices, and the unknown contribution of physiological and device-related delay [71].

Difference between CGM reading and reference measurement, as expressed by MARD, was in some subcutaneous devices as high as 30%, i.e., in the FreeStyle Libre [48]. This device, however, is developed for outpatient use. It applies to the definition of CGM since it measures interstitial glucose every 15 min, but it has no alarm functions, and lacks the possibility to manually calibrate (since it is factory-calibrated). The MARD of the intravascular devices was overall lower than of subcutaneous devices. This might in part be due to the systematic error that is introduced since calibration of interstitial positioned devices is performed with blood glucose levels. Moreover, the MARD depends highly on the reference method, which has its own error, as well as the possible delay between actual blood samples and reported time of analysis. Meanwhile, even when MARD is higher than recommended, the provided trend information could still be beneficial in terms of early detection and treatment of hypo- and hyperglycemia. Not all studies gave a clear description of reference sample location and measurement device. It is known that the MARD is considerably lower in hypoglycemic ranges. However, included studies all reported a low number of hypoglycemic events, due to adequate glucose algorithms. Thereby we cannot conclude on accuracy in the hypoglycemic area. 

Bland-Altman plots show the difference between the CGM reading and the reference, plotted against their mean [34]. Overall, the mean bias was low, indicating a low systematic error, but there are wide limits of agreement, indicating high random errors. These random errors could be due to both the CGM device as patient specific factors. 

The ISO criteria are originally used to determine whether a device is accurate enough to be marketed commercially for outpatient use. The 2003 criteria state that 95% of the readings should be within 20% of the reference measurement. In 2013 the accuracy limits were revisited to at least 95% of the reading within 15% of the reference. Only some intravascular devices met this criteria [52,54,68,72]. By requiring 95% of measured values to meet this criterion, still 5% of the measurements can differ from the reference by any amount. This might be acceptable in home-use, for which these meters were designed, but seems potentially dangerous in ICU setting. 

The majority of the included studies used the CEG to assess the clinical accuracy of the CGM system. CEG categorizes pairs in terms of the consequence of treatment decisions. It was initially designed for evaluation of self-monitoring devices, for which 95% of the pairs should fall in zone A [33]. Rate and direction, two important features are not taken into account in the original CEG. For this, modified (continuous) error grids have been developed, but their value in the assessment is questionable [71]. In most studies the majority of data pairs were in the acceptable zones A and B, especially with the intravascular devices. However, there were quite some studies reporting a certain percentage of pairs in the dangerous D and E zones. The targets used in the original CEG are 70 to 180 mg/dL, but, as shown in the table, most ICUs use different targets, but do not adjust this in their CEG. 

Therefore, overall the intravascular devices show better accuracy than the subcutaneous devices, possibly because the former are not sensitive for disturbances in microcirculation and they have no lag time to consider. Most of the subcutaneous devices were originally designed for home-use and thereby not equipped to deal with critical ill patients. However, 80% of the articles studying intravascular devices included only elective (cardio surgical) patients. Considering that these patients are not as ill as the general ICU population, external validity is limited. In addition, most subcutaneous devices were studied in the general ICU population. Subcutaneous devices are less invasive, with a lower risk for disturbance by infused medication and glucose solutions. The Sentrino CGM system is the only subcutaneous device primarily designed for use in critically ill patients. It was studied in three included studies, of which in two it did not perform with satisfactory accuracy [55,56]. It is not clear what the effect of rapid glucose changes, severity of illness and interference of medication (e.g., vasopressin, acetaminophen) is on the accuracy of both types of CGM devices, since this was not directly investigated. Not surprisingly, the accuracy of subcutaneous systems markedly improves when performing more frequent calibration than advised by the manufacturer for outpatient use [37,50,56,57]. In the study by Leelarathna et al., this meant one calibration per 2.5 to 3.5 h. Thus, some of one of the advantage of continuous glucose monitoring, reducing the amount of required blood samples, and reduced time spent on blood glucose control, is partly lost when calibrating more frequently. It is recommended that CGM systems should ideally not need more than 3 calibrations every 24 h at the ICU [31]. As long as a CGM system needs calibration with blood glucose measurements, it will be difficult to truly replace this. Optiscanner is the only (intravascular) device that does not need calibration. 

Adverse events of subcutaneous devices seemed rare, and consisted of the worst case of minor bleeding [55], bruises or redness [56]. With the intravascular devices, thrombosis was described, especially with the intravenous devices [53], leading to one case of device related thrombosis. Overall, the adverse event rate in all studies was low, and no serious adverse events were reported. Thereby, adverse effects will not limit the use of CGM systems in the intensive care unit. 

Only one study by Boom et al. investigated the effect of a subcutaneous CGM system on workload and costs, and showed that using these systems significantly improved both these outcomes [38]. However, the timeframe was relatively short (24 h), and the cost benefit was small, so this requires further investigation. It did show that the use of CGM systems, which are quite expensive, is not a priori expense. CGM did improve nursing workload. The advantage of using a continuous monitor is mainly determined by the reduced number of point-of-care measurements, which are time intensive. However, CGM systems do require regular calibrations to achieve a certain accuracy, which carry their own workload. Theoretically, a totally automated closed-loop system that does not need to be calibrated will be able to bring the workload of glucose control to a minimum. 

In general, CGM has potential benefits in the ICU, such as improvement of time in target, reduction of glycemic variability and less staff workload. However the current evidence on the use of CGM systems in critically ill patients is not sufficient. Large randomized trials have not yet been performed, especially not with use of an adapted glucose algorithm for continuous data. Thus, we lack sufficient data to draw conclusions on the clinical benefit, although the available evidence seems to point in a slightly favorable direction, mainly because of less hypoglycemic events and especially when combined with an adequate glucose algorithm. CGM accuracy seems moderate to good with intravascular devices, but the majority of studies was limited to cardiac surgery patients. The accuracy of both subcutaneous and intravascular devices might be adequate enough to guide alarms, but when CGM is used to guide therapy, it might require improvement. Accuracy metrics lack standardization and do not seem tailored for the assessment of CGM in critically ill patients, despite the recently made consensus statement. We emphasize the importance of standardization of the assessment methods and future research on accuracy and efficacy in these patients. Ultimately, this may lead the development of a true closed-loop glucose control system.

## 5. Conclusions

There is sparse evidence for the effect of CGM systems in critically ill patients compared to standard blood glucose measurement, with only five RCTs assessing the impact of subcutaneous devices on glycemic control. Overall, CGM systems do not seem to clearly improve glycemic control in a clinically significant manner. Only when incorporated into a fully automated closed loop the mean glucose and time in the target range did improve in a single trial. In two trials hypoglycemia decreased with the use of CGM, and one trial showed decreased nursing workload. The accuracy of intravascular devices seems better than subcutaneous devices, at the cost of some risk for adverse events like thrombus formation. Intravascular devices are however assessed in a relative small number of studies, and mainly in cardiac surgery patients. The reported accuracy metrics of both subcutaneous and intravascular devices differ widely among studies and a clear definition of assessment methods is limited to an expert consensus statement. Safety in terms of local complications is good, but there is a potential danger as a consequence of inaccurate measurements, making improvements desirable. However, theoretically CGM systems still have the potential to improve glycemic control, especially when technically improved or combined with an appropriate glucose algorithm adapted to continuous measurements. More robust data is needed, preferably from larger multi-center studies with head-to-head comparisons, to demonstrate beneficial effect on both outcome (glycemic control, length of stay, mortality) and costs. 

## Figures and Tables

**Table 1:** Descriptives of the included articles by their main outcome (*n* = 37).

Main Outcome	Accuracy (*n* = 32)	Effectivity (*n* = 5)
**Year of publication (range)**	2006–2016	2010–2015
**Study design (*n*, %)**		
RCTs	1 (3.1%)	5 (100%)
Observational trial	30 (93.8%)	0
Pooled analysis of two RCTs	1 (3.1%)	0
**Type of patients (*n*, %)**		
Mixed ICU patients	12 (37.5%)	3 (60.0%)
Medical patients	0	0
General surgical patients	5 (15.6%)	0
Cardiac surgery patients	9 (28.1%)	1 (20.0%)
Neurosurgical patients	2 (6.3%)	1 (20.0%)
Children	4 (12.5%)	0
**Maximum study duration (hours)**		
Median [IQR]	72 [48–72]	72 [36–108]
Range	24–168	24–120
Not reported	4 (12.5%)	0
**Number of analysed patients**		
Median [IQR]	23 [19–48]	35 [24–140]
Range	8–174	24–156
**Type of CGM device studied (*n*, %)**		
Subcutaneous	19 (59.4%)	5 (100%)
Intravascular	10 (31.3%)	0
Transdermal	1 (3.1%)	0
Subcutaneous and intravascular	2 (6.3%)	0
**Reference measurement (*n*, %)**		
Arterial	21 (65.6%)	5 (100%)
Venous	4 (12.5%)	0
Arterial and venous	4 (12.5%)	0
Not described	3 (9.4%)	0
**Number of paired samples (*n*, %)**		
Median [IQR]	672 [346–1028]	440 [277–603]
Range	34–2045	277–635
Not reported	2 (6.3%)	2 (40%)

Percentages are based on the total amount of 37 articles. Due to rounding percentages might not sum up to 100%. Abbreviations: ICU, Intensive Care Unit; IQR, interquartile range; *n*, number; RCT, randomized controlled trial.

**Table 2:** Reported effectivity outcomes of randomized controlled trials assessing subcutaneous CGM systems (*n* = 5).

				Findings								
CGM System	*N* ^a^	Study Population		Average Glucose ^b^ (mg/dL)		Time in Range ^c^ (%)		Time in Hypogly-Cemia ^c^ (%)		Time in Hypergly-Cemia ^c^ (%)		Target Glucose Range ^d^ (mg/dL)
GlucoDay, A. Menarini Diagnostics	35	Mixed ICU population	Intervention	119.0	±17.0	37.0	±12.0	0.6	±1.6	4.0	±5.0	80–120
			Control	122.0	±11.0	34.0	±10.0	2.4	±4.3	2.0	±3.0	
FreeStyle Navigator I, Abbott	156	Mixed ICU population	Intervention	127.9	±19.8	75.0	±18.0		3 episodes in 3 patients	3.0	±7.0	90–160
			Control	135.1	±23.4	71.0	±20.0		4 episodes in 4 patients	4.0	±9.0	
FreeStyle Navigator I, Abbott	24	Neurosur-gical patients	Intervention	142.3	[133.3–147.7]	54.3 *	[44.1–72.8]	0.0			1 episode in 1 patient	110–140
			Control	164.0	[149.6–234.2]	18.5 *	[0.1–39.9]	0.0			11 episodes in 5 patients	
Guardian REAL-Time, Medtronic	24	Cardiosur-gical patients	Intervention	111.7	±1.8	46.3	±5.5		0 episodes			80–110
			Control	109.9	±10.8	46.2	±6.5		2 episodes			
Guardian REAL-Time, Medtronic	124	Mixed ICU patients	Intervention	105.8	±18.1	59.0	±20.4		1.6% of patients *			80–110
			Control	110.6	±10.4	55.0	±18.0		11.5% of patients *			

Values are displayed as mean ± SD or median [IQR]. ^a^ Number of analysed patients; ^b^ Average glucose levels are based on the sensor measurement of the CGM system; ^c^ The time in different ranges is dependent of the predefined ranges of the different studies and could thereby differ among studies; ^d^ Ranges are when necessary converted into mg/dL, and rounded to dozens. * Indicates a statistically significant difference between the intervention and control group on the reported outcome. Abbreviations: CGM, continuous glucose monitoring; *N*, number (of patients).

**Table 3:** Reported accuracy outcomes of the included studies that assessed subcutaneous continuous glucose monitoring (CGM) systems (*n* = 26).

				Findings										
Author Year	CGM System	*N* ^a^		MARD ^b^ (%)		ISO ^c^ (%)	Clarke Error Grid ^d^ (% in Zones A–E)					Bland-Altman ^e^ (mg/dL)	Target Range ICU ^f^ (mg/dL)	Reference Sample
							A	B	C	D	E			
**Adults**														
Wollersheim et al., 2016	Sentrino, Medtronic	532 (20)		15.3	(13.5–17.0)	76.9	76.9	21.6	0.2	0.9	0.4	0.5 (−63.5 to 64.6)	80–149	Arterial or venous
Schierenbeck et al., 2016	FreeStyle Libre, Abbott ***	578 (26)		30.5	±12.4	7.0	18.9	80.2	0.9			−43.2 (−82 to −4.5)	90–180	Arterial
v. Hooijdonk et al., 2015	Unspecified, Medtronic	929 (50)		14.8		75.8	75.3	23.5	0.3	0.9	0.0	−0.6 (−57.2 to 56.0)	90–144	Arterial
Song et al., 2015	Guardian REAL-Time, Medtronic	331 (22)	Thigh	23.7	±30.2		60.1	34.4	3.3	1.5	0.6	6.6. (−109.7 to 122.9)		Arterial
		270 (22)	Abdomen	23.2	±19.5		57.0	36.7	3.0	3.3	0.0	14.9 (−108.2 to 138.1)		
Sechterberg et al., 2015	FreeStyle Navigator I, Abbott	183 (8)		11.1	±8.3	84.2						−8.0 (−49.7 to 33.8)	90–160	Arterial
De Block et al., 2015	GlucoDay, A. Menarini Diagnostics	635 (35)		11.2		87.0	87.1	11.5	0.4	1.0	0.0		80–120	Arterial
Saur et al., 2014	Symphony, Echo Therapeutics *	570 (15)		12.3			81.7	18.3	0.0			7.8 (−31.5 to 47.2)	100–180	Arterial
Leelarathna et al., 2014	FreeStyle Navigator I, Abbott	516 (12)	Enhanced calibration	9.6	±8.9	87.8	87.8	12.2	0.0			−1.8 (−12.6 to 7.2)		Arterial
		544 (12)	Normal calibration	15.6	±12.0	70.2	70.2	29.0	0.0	0.8	0.0	−19.8 (−41.4 to 1.8)		
Kosiborod et al., 2014	Sentrino, Medtronic	870 (21)		12.8	(11.9–13.6)		83.0	16.0	0.8	0.0		2.5 (−43.7 to 48.7)	<140	Venous
Boom et al., 2014	FreeStyle Navigator, Abbott	440 (177)		13.7 *	[8.0–23.0]								90–160	Arterial
Aust et al., 2014	CGMS System Gold, Medtronic	342 (10)					86.3	12.9	0.0	0.9	0.0	0 (limits not reported)	80–150	Arterial
Yue et al., 2013	DGMS, San MediTech	314 (18)		14.4	±12.2		74.8	25.2	0.0			1.8 (−59.5 to 63.1)	140–200	Venous
Siegelaar et al., 2013	Guardian REAL-Time, Medtronic	(60)		14.0	[11.0–18.0]		73.2	25.2	1.3				90–144	Arterial
	FreeStyle Navigator I, Abbott			11.0	[8.0–16.0]		81.8	17.7	0.5		0.0			
Leelarathna et al., 2013	FreeStyle Navigator I, Abbott	(27)		7.0*	[3.5–13.0]	87.8							110–180	Arterial
Kopecky et al., 2013	Guardian REAL-Time, Medtronic	277 (24)					66.4	31.1	0.0	2.5	0.0		80–110	Arterial
Lorencio et al., 2012	Unspecified, Medtronic	956 (41)		13.5	(6.0–24.1)	68.1						6.4 (−53.1 to 65.8)	120–160	Arterial
Siegelaar et al., 2011	Guardian REAL-Time, Medtronic	1017 (60)		14.0	[11.0–17.0]									Arterial
	FreeStyle Navigator I, Abbott			10.0	[8.0–16.0]									
Brunner et al., 2011 **	Unspecified, Medtronic	2045 (177)		7.3	(6.8–7.8)	92.9	99.1		0.5	0.4	0.0	2.0 (−21.0 to 25.0)		Arterial
Rabiee et al., 2009	Unspecified, Dexcom	84 (19)					75.0	25.0	0.0				90–120	Unknown
Holzinger et al., 2009	CGMS System Gold, Medtronic	736 (50)				94.0	98.6		0.0	0.7	0.7	0.7 (−1.4 to 2.9)		Arterial
De Block et al., 2006	GlucoDay, A. Menarini Diagnostics	820 (50)	2-pt calibration				72.5	22.2	4.5	0.7	0.1		110–140	Arterial
		555 (50)	6-pt calibration				80.5	16.2	1.6	1.4	0.2			
Corstjens et al., 2006	CGMS System Gold, Medtronic	165 (19)					87.3	12.7	0.0			1.8 (−41.4 to 36.9)	110–140	Arterial
**Children**														
Piper et al., 2006	CGMS System Gold, Medtronic	246 (20)		17.6			66.3	32.5	0.0	1.2	0.0			Arterial
Branco et al., 2010	CGMS System Gold, Medtronic	34 (14)		23.0			53.0	47.0	0.0					Arterial
Bridges et al., 2010	Guardian REAL-Time, Medtronic	1555 (47)		15.3			74.6	23.3	2.1		0.0	−1.5 (−59.5 to 56.5)		Unknown
Phrabhudesai et al., 2015	Guardian REAL-Time, Medtronic (Enlite sensor)	235 (19)		17.3 *			66.0	28.5	0.0	7.2	0.0	−5.1 (−76.8 to 66.6)		Unknown

^a^ Total number of paired samples, in parenthesis the number of included patients; ^b^ MARD is reported with its corresponding 95% confidence interval, SD (±), or [IQR]. Reported MARD is of the entire glycemic range; ^c^ Percentage of measurements >75 mg/dL that are within 20% of the reference measurement (ISO15197:2003); ^d^ Clarke error grid reports the percentage of measurements in zones A to E; ^e^ Bland-Altman analysis is reported as mean bias (limits of agreement); ^f^ Ranges are when necessary converted into mg/dL, and rounded to dozens. * Indicates a median ARD instead of a MARD. ** Combined analyses of Holzinger, 2009 and Holzinger, 2010. *** Patients received both the subcutaneous FreeStyle Libre and the intravascular Eirus System (results on the latter are in Table 4). Abbreviations: CGM, continuous glucose monitoring; CI, confidence interval; ISO, international organization for standardization; MARD, mean absolute relative difference; *N*, number (of patients).

**Table 4:** Reported accuracy outcomes of the included studies that assessed intravascular CGM systems (*n* = 11).

				Findings										
Author Year	CGM System	*N* ^a^		MARD ^b^ (%)		ISO ^c^ (%)	Clark Error Grid ^d^ (% in Zones A–E)					Bland-Altman ^e^ (mg/dL)	Target Range ICU ^f^ (mg/dL)	Reference Sample
							A	B	C	D	E			
Schierenbeck et al., 2016	Eirus System, Maquet Critical Care *	514 (26)		6.5	±8.2	90.0	94.0	6.0	0.0			0.9 (−27.0 to 29.0)	80–149	Arterial or venous
Nohra et al., 2016	Optiscanner 5000, Optiscan	347 (24)		8.0	(7.3–8.7)		94.8	5.2	0.0			−5 (−28 to 18)		Unknown
Leopold et al., 2016	Eirus System, Maquet Critical Care	594 (12)		7.5		93.6	93.6	6.4	0.0			4.1 (−20.5 to 28.6)	90–144	Arterial
Strasma et al., 2015	Glucath, Medtronic	1799 (70)	Arterial sensor	9.6		89.4						−2.1 (−34.5 to 29.6)	100–180	Arterial or central venous
		1799 (70)	Venous sensor	14.2		72.2						−6.5 (−53.8 to 39.8)		
Macken et al., 2015	GluCath, Medtronic	758 (20)		6.4		97.0						−10.8 (−466.2 to 446.4)		Arterial
Crane et al., 2015	GlySure, GlySure	(33)	Cardiac surg. patients	9.9			88.2	11.8						Venous
		(14)	General patient	8.0			95.0	5.0						
Bochiccio et al., 2015	IVBG System, Edwards Lifesciences	996 (100)		8.2	±10.5	93.3	93.2	5.8	0.2	0.8	0.0			Arterial or venous
Foubert et al., 2014	GlucoClear, Edwards Lifesciences	1093 (10)		5.1		99.4	99.4	0.6	0.0			−3 (−15.6 to 9.6)	80–110	Venous
Flower et al., 2014	GluCath, Medtronic	437 (21)		13.0		80.8						−5.8 (−54.5 to 42.9)		Arterial
Schierenbeck et al., 2013	Eirus System, Maquet Critical Care	607 (30)		5.6		97.2	97.0	3.0	0.0			−2.2 (−14.8 to 10.5)		Arterial
Schierenbeck et al., 2012	Eirus System, Maquet Critical Care	994 (50)		5.0		99.2	99.0	1.0	0.0			0.4 (−19.5 to 22.0)		Arterial and venous

^a^ Total number of paired samples, in parenthesis the number of included patients; ^b^ MARD is reported with its corresponding 95% confidence interval, SD (±), or [IQR]. Reported MARD is of the entire glycemic range; ^c^ Percentage of measurements >75 mg/dL that are within 20% of the reference measurement (ISO15197:2003); ^d^ Clarke error grid reports the percentage of measurements in zones A to E; ^e^ Bland-Altman analysis is reported as mean bias (limits of agreement); ^f^ Ranges are when necessary converted into mg/dL, and rounded to dozens. * Patients received both the intravascular Eirus System and the subcutaneous FreeStyle Libre (results on the latter are in Table 3). Abbreviations: CGM, continuous glucose monitoring; CI, confidence interval; ISO, international organization for standardization; MARD, mean absolute relative difference; *N*, number (of patients).

## Appendix A. Ovid MEDLINE Search

Database(s): Epub Ahead of Print, In-Process & Other Non-Indexed Citations, Ovid MEDLINE(R) Daily and Ovid MEDLINE(R) 1946 to Present Search Strategy: 26 July 2016.

**#**	**Searches**	**Results**
1	((glucose or BG) adj1 (sensor* or biosensor* or continuous* or realtime or real time)).tw,kf.	5099
2	((glucose or BG) adj monitor*).tw,kf.	5227
3	((continuous* or real time or subcutan* or arter* or venous or intravasc*) adj monitor* adj12 glucos*).tw,kf.	325
4	(continuous adj3 (glucose measurem* or glucosemeter*)).tw,kf.	93
5	(CGM or CGMs or GCMS* or RTCGM* or BGM or BGMs or CIGM* or IVCGM* or scCGM* or sCGM* or tCGM* or MDCGM* or IACGM).tw,kf. and glucose.mp.	1543
6	(IVBG or (intraven* adj (blood glucose or BG) adj3 system*)).tw,kf.	4
7	(GlucoDay* or Freestyle or Libre or Navigator or Medtronic* or MiniMed* or Sentrino* or Enlite* or Optiscanner or Eirus* or Glucath* or Glysure* or Symphony or Glucoclear* or (DexCom adj1 STS) or (Guardian adj2 real time)).tw,kf. and glucose.mp.	552
8	clarke error grid.tw,kf.	184
**9**	**or/1−8 [CGM]**	**8143**
10	animals/ not humans/	4,248,414
**11**	**9 not 10 [human CGM]**	**7501**
12	critical care/or exp life support care/or subacute care/or intensive care units/or critical illness/or burn units/or coronary care units/or recovery room/or respiratory care units/[MESH]	105,986
13	((intensive or critical*) adj (care or ill*)).tw,kf.	145,729
14	(severe* adj (burn* or ill)).tw,kf.	8464
15	((coronary or cardiac) adj2 care).tw,kf.	8404
16	(((acute care or respiratory care or acute stroke or burn*) adj unit*) or acute stroke care).tw,kf.	3140
17	(trauma adj2 (cent* or unit*)).tw,kf.	13,030
18	recovery room*.tw,kf.	2924
19	emergency medicine.tw,kf.	11,055
20	(intensivmed* or (intensive and care)).jw,ot.	25,004
21	(ICU or ICUs or CCU or CCUs or MICU or MICUs or CVICU* or SICU or SICUs or BICU or BICUs).tw,kf.	43,021
22	(ECMO or ECLS or (extracorporeal adj3 (circulation or circuit or bypass* or life support* or ventricular assist*))).tw,kf.	14,712
**23**	**or/12−22 [ICU]**	**261,652**
**24**	**11 and 23 [CGM + ICU]**	**370**
**25**	**remove duplicates from 24**	**356**

## Appendix B. Overview of Important Assessment Tools to Evaluate Point Accuracy of Continuous Glucose Monitoring (CGM) Systems

**Tool**	**Definition**	**Strengths**	**Weaknesses**	**ICU recommendations**
Mean Absolute Relative Difference (MARD)	Percentage difference between CGM sensor reading and a value measured at the same time using a reference method. Derived as ([sensor-reference]/reference) × 100%.	Easy to compute and interpret, can be computed in different glucose range.	No distinction between positive and negative or systematic and random errors. Affected by glucose values and study design. Often unclear whether MARD or median absolute relative difference is computed.	Acceptable when <14% [73], >18% indicates poor accuracy [31].
ISO 15197 guideline (2003)	Percentage CGM sensor readings within 15 mg/dL from the reference when the blood glucose is ≤75 mg/dL or within 20% from the reference when the blood glucose is >75 mg/dL.	Simple.	Does not take rate of glucose change and temporal order of the measurements into account. Resting 5% can differ by any amount.	≥98% within 12.5% of a reference standard (or within 10 mg/dL for reading <100 mg/dL), remaining 2% within 20% [31].
ISO 15197 guideline (2013)	Percentage CGM sensor readings within 15 mg/dL from the reference when the blood glucose is ≤100 mg/dL or within 15% from the reference when the blood glucose is >100 mg/dL.			
Clarke error grid	Pairs CGM sensor readings with reference measurements, and categorizes pairs in terms of the consequence of treatment decisions. **Zone A** Within 20% of the reference value, clinically accurate. **Zone B** >20% difference from the reference value, benign errors since it will not lead to inappropriate clinical decisions. **Zone C** Overcorrection errors, unnecessary but harmless corrections. **Zone D** Dangerous failure to detect hypo- or hyperglycemia. **Zone E** Erroneous treatment error (opposite of intended treatment).	Simple. Indicates clinical significance by showing the implication on therapy.	Developed for capillary blood glucose testing systems. Original grid was designed with an arbitrary target range of 70−180 mg/dL and assumes no change in treatment when readings lie within that range. No allowance for the rate at which blood glucose concentration is changing or the frequency with which the blood glucose concentration is being measured.	100% in zone A + B, favorably in zone A [74].
Bland-Altman plot	Plot of the reference measurement or average of the two (*x*-axis) against the difference between CGM system and reference measurement (*y*-axis). Reported as mean bias with upper and lower limits of agreement (mean bias ± 1.96 × SD). Represents the random variation around the mean bias.	Simple. Possibility to distinct between systematic and random error.	Does not allows for the effect of different ranges and trend.	No recommendations.
